# A “one-step” treatment for symptomatic lead-related venous obstruction using percutaneous lead extraction, venous stenting, and new device implantation

**DOI:** 10.1016/j.hrcr.2024.03.004

**Published:** 2024-03-15

**Authors:** Tsuyoshi Isawa, Takehiro Nomura, Taku Honda, Kazuhiro Yamaya, Shigeru Toyoda

**Affiliations:** ∗ Department of Cardiology, Sendai Kousei Hospital, Sendai, Japan; † Department of Cardiovascular Surgery, Sendai Kousei Hospital, Sendai, Japan; ‡ Department of Cardiovascular Medicine, Dokkyo Medical University, Mibu, Japan

**Keywords:** Cardiac resynchronization therapy-defibrillator, Lead extraction, Tandem approach, Venous obstruction, Venous stenting

**Key Teaching Points**

• A “one-step” percutaneous intervention for symptomatic lead-related venous obstruction is feasible in a patient with cardiac resynchronization therapy-defibrillator (CRT-D), consisting of percutaneous lead extraction using a tandem approach, venous stenting, and immediate implantation of a new CRT-D through the same access site in a single session.
• The tandem approach allows a lead to serve as a guiderail for a powered sheath through a venous occlusion, thus gaining access to a cardiac chamber.
• Venous stenting is important in the endovascular management of symptomatic lead-related venous obstruction to avoid recoil. However, jailing the lead between the vein wall and stent should be avoided.

## Introduction

The global prevalence and intricacies of cardiovascular implantable electronic device (CIED) implantations have been on the rise, especially with introduction of cardiac resynchronization therapy-defibrillator (CRT-D).^1^^,^^2^ However, this advancement in procedures has been accompanied by a corresponding increase in the rate of complications. In particular, symptomatic lead-related venous obstruction is a serious complication affecting 1 out of every 20 patients with CIEDs.^3^ It can cause facial and upper extremity edema, as well as blood removal failure in patients on hemodialysis. This condition can be treated by anticoagulation, percutaneous lead extraction, endovascular treatment, and open surgical reconstruction. However, data-driven guidance for its management, including the manner of intervention, is lacking. Herein, we present a case of symptomatic lead-related venous obstruction managed with a multidisciplinary approach consisting of percutaneous extraction of the CRT-D system using a tandem approach,^4^ venous stenting at the site of occlusion, and immediate implantation of a new CRT-D via the same access site in the same procedure.

### Case report

**Figure 1:**
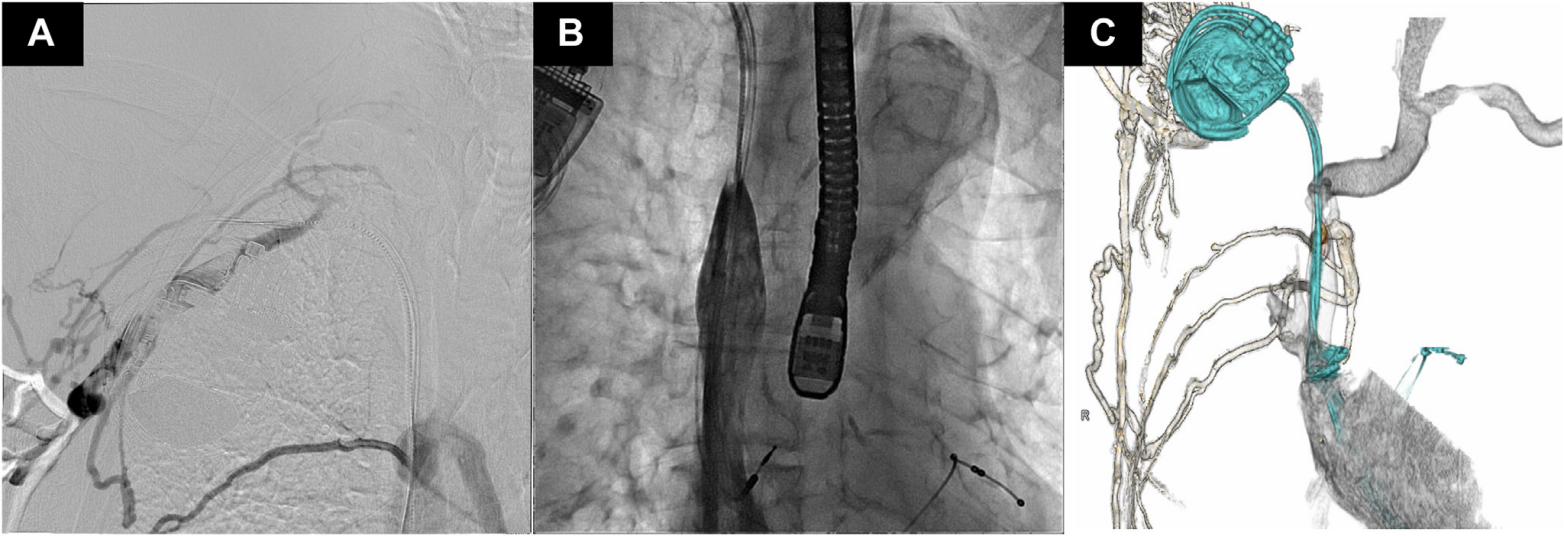
Preprocedural images. A: Digital subtraction venography showing the occluded right brachiocephalic vein and upper portion of the superior vena cava. B: Retrograde venography showing the occluded upper superior vena cava. C: Computed tomography revealing communication between the right axillary and the azygos veins via the intercostal veins. The azygos vein became the main collateral, draining into the superior vena cava.

A 76-year-old male patient on chronic hemodialysis with an arteriovenous hemodialysis shunt on his left arm presented with progressive edema in the upper body that began 2 months earlier. He also experienced blood removal failure lasting for 2 months. Eight years prior, he underwent CRT-D implantation on the right side because of medication-refractory heart failure (New York Heart Association [NYHA] functional class III and left ventricular ejection fraction of 23%) and evidence of conduction disturbances (QRS width 203 ms) with a left bundle branch block. Imaging studies showed obstruction of the right brachiocephalic vein and upper superior vena cava (SVC) with drainage via the azygos system (Figure 1 and Supplemental Video 1). The patient was diagnosed with symptomatic lead-related venous obstruction. After discussion with his heart team, a multidisciplinary approach was adopted with percutaneous lead extraction, venous stenting, and new CRT-D system implantation in a single session. This was decided because he positively responded to CRT-D (NYHA functional class I and left ventricular ejection fraction of 52% on this admission) and had an arteriovenous hemodialysis shunt on the left side.

Percutaneous lead extraction was performed in a hybrid operating room under general anesthesia. The pocket was widened, and the leads were dissected from the adhesions inside the pocket. Lead extraction of the left ventricular lead (Attain Performa Straight 4398–78; Medtronic, Minneapolis, MN) was initiated via the superior approach using a 9F Evolution Shortie RL sheath (Cook Medical, Bloomington, IN), but it did not advance past the right brachiocephalic vein occlusion. The target lead was switched from the left ventricular lead to the atrial lead (CapSure Z NOVUS 5554–53; Medtronic). Unfortunately, after dissection of the adhesion around the atrial lead at the right brachiocephalic vein, it was completely extracted before catching the atrial lead via the femoral approach (Supplemental Video 2). Consequently, a 0.035-inch guidewire did not cross through the occluded vein. Thus, a tandem approach was applied by catching and pulling the lead femorally before the superior approach. This was done to ensure that the 0.035-inch guidewire crossed though the occluded vein inside an Evolution sheath.

**Figure 2:**
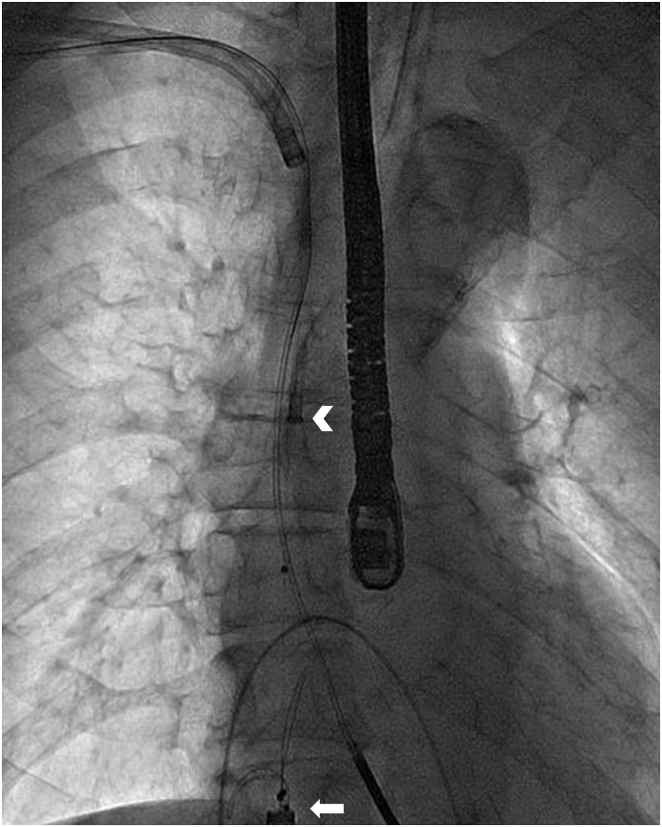
A tandem approach. A 13 mm Needle’s Eye Snare (Cook Medical, Bloomington, IN) (*white arrow*) was employed femorally to grasp the targeted lead and provide countertraction as the 9F Evolution RL sheath (Cook Medical) (*white arrowhead*) with an outer sheath was advanced over the targeted lead through a superior access.

After securing of the left ventricular lead via the femoral approach using a 13 mm Needle’s Eye Snare (Cook Medical), a superior approach was started with a 9F Evolution RL sheath (Figure 2 and Supplemental Video 3), which was able to advance into the right atrium through the occluded SVC. The lead was completely extracted, and a 0.035 inch guidewire was advanced through the Evolution RL sheath into the right atrium, maintaining venous access across the occluded vein. Likewise, after securing of the right ventricular lead (Sprint Quattro 6935M–55; Medtronic) using the Needle’s Eye Snare, an 11F Evolution Shortie RL sheath was advanced until the right atrium (Supplemental Video 4). Afterward, the right ventricular lead was completely extracted, and another 0.035 inch guidewire was advanced through the Evolution sheath into the right ventricle. Endovascular treatment was performed following lead extraction. A guidewire was advanced in a retrograde fashion from the right femoral vein into the right subclavian vein across the SVC. The right brachiocephalic vein and SVC were predilated with a 7.0 × 40 mm Mustang balloon (Boston Scientific, Marlborough, MA). We successfully implanted a 14 × 60 mm SMART stent (Cordis Corp, Miami Lakes, FL) and a 14 × 60 mm Luminexx stent (BARD, Inc, Murray Hill, NJ), extending from the right brachiocephalic vein into the SVC. Postdilatation was accomplished with a 7.0 × 40 mm Mustang balloon at each site. Poststenting venogram showed improvements in SVC expansion and blood flow (Supplemental Video 5).

**Figure 3:**
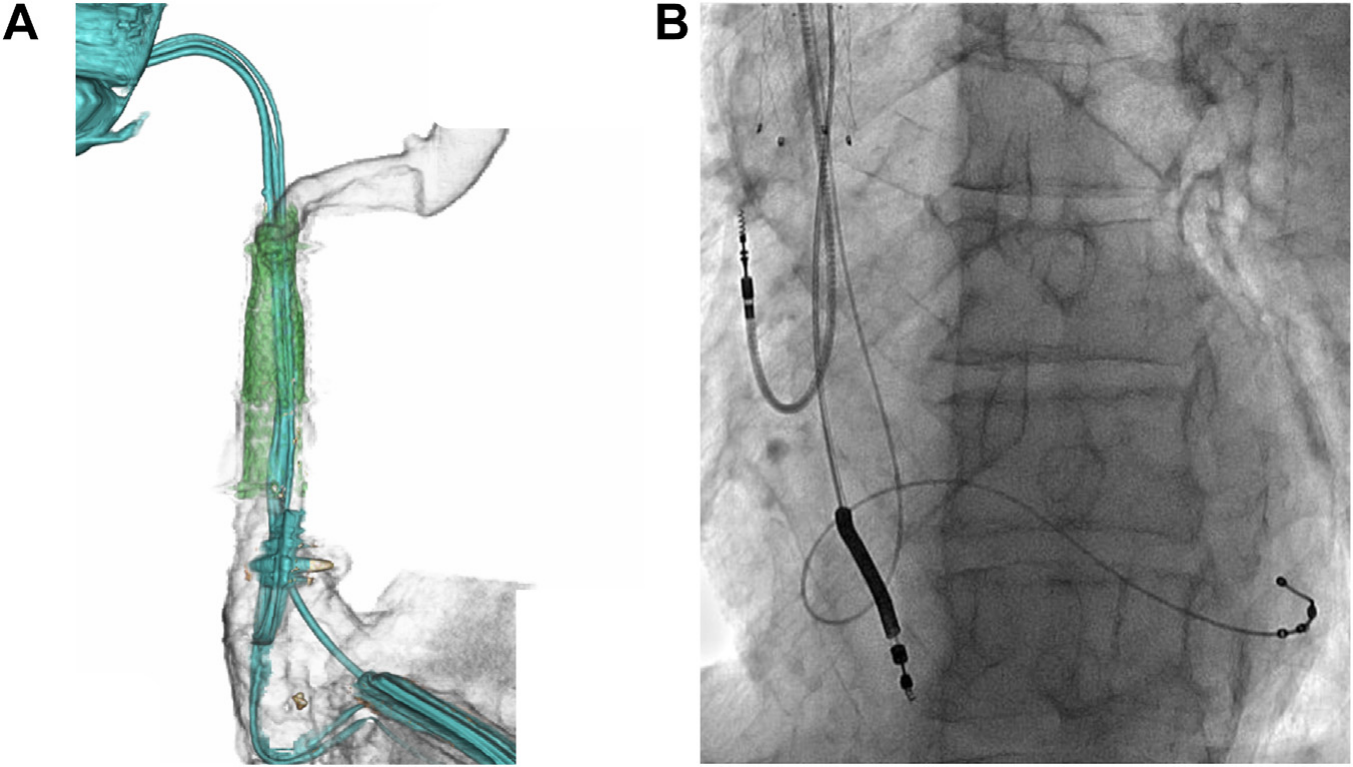
Postprocedural images. A: Computed tomography showing improved expansion of the right brachiocephalic vein and superior vena cava with stents. B: Fluoroscopic image showing a new cardiac resynchronization therapy-defibrillator system, with leads advanced through the newly stented veins (left anterior oblique view).

**Supplementary Figure 1:**
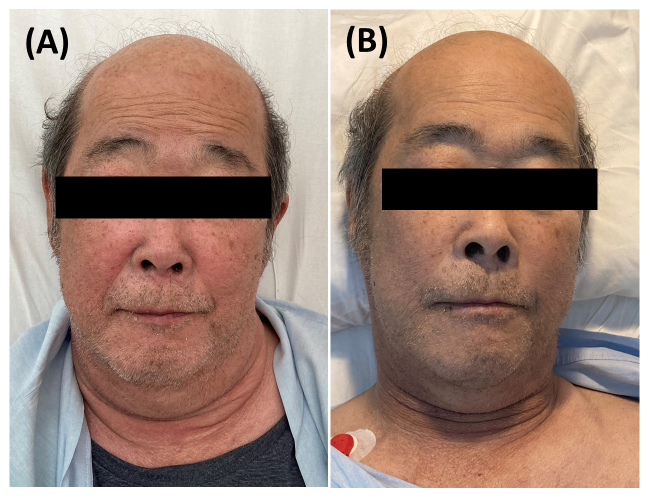
A comparison between front images of the patient taken before lead extraction (A) and two weeks after lead extraction with venous stenting (B). Post-lead extraction image revealing that the previously noted facial plethora and neck fullness have resolved.

At the start of CRT-D reimplantation, the 2 jailed guidewires from the right side were pulled back into the right subclavian vein and recrossed into the right atrium through the intravenous stents. The 1-puncture 2-sheath technique was used to introduce 2 peel-off sheaths into the right subclavian vein, because only 2 guidewires were crossed through the right subclavian vein from the right side. Three venous access sites were obtained, and the new CRT-D was successfully implanted. None of the antimicrobial-impregnated CIED envelopes were used because they were not available at our hospital. Postprocedural imaging revealed a patent SVC, with the 3 leads of new CRT-D via the right subclavian vein advanced through the newly stented veins (Figure 3). Antiplatelet treatment with aspirin was prescribed to prevent thrombosis. Clinically, the symptoms and signs of venous obstruction resolved (Supplemental Figure 1), and the patient received conventional hemodialysis (3 sessions/week, 3–6 hours each session) starting on the day after the procedure.

## Discussion

In this report, we presented a multidisciplinary approach for the treatment of symptomatic lead-related venous obstruction. We performed percutaneous extraction of the entire CRT-D system using a tandem approach, venous stenting at the site of occlusion, and new CRT-D device implantation with the same venous access in a single session. This approach was effective for treating symptomatic venous obstruction secondary to the CRT-D system. This case raises 2 important clinical issues. First, a “one-step” percutaneous approach for symptomatic lead-related venous obstruction was feasible in a patient with CRT-D. Second, the tandem approach allowed the lead to serve as a guiderail for the powered sheath through the venous occlusion, allowing us to access the cardiac chamber.

The feasibility of a “one-step” percutaneous approach for symptomatic lead-related venous obstruction was first described in patients using pacemaker devices,^5^ but no reports have discussed its feasibility in patients with CRT-D. This is the first known description of percutaneous lead extraction followed by venous stenting and reimplantation of CRT-D. A “one-step” approach including immediate implantation of a new CRT-D via the same access site (ie, contralateral to an arteriovenous hemodialysis shunt) in the same procedure was necessary for our patient, because venous obstruction induced by device implantation on the ipsilateral side to an arteriovenous hemodialysis shunt can cause blood removal failure in the future.

The tandem approach was effective for maintaining venous access, and its benefits are most evident among patients with leads of the CRT-D system because 3 venous access sites are required. In a previous report on percutaneous lead extraction for symptomatic SVC syndrome,^6^ powered sheaths and guidewires failed to cross the venous obstruction in 5 out of 16 patients, because the superior approach alone was used in most of the patients. In contrast, the tandem approach makes it easier for the powered sheath to pass through the venous obstruction site; this enables a guidewire to transverse the venous obstruction into the cardiac chamber through the powered sheaths after the lead is extracted.

Stent placement plays an important role in the endovascular treatment of venous obstruction to avoid acute recoil. Several studies have reported good long-term efficacy with stent placement.^7^, ^8^, ^9^ Moreover, venous reocclusion has also been reported in a patient with SVC syndrome treated with balloon venoplasty alone.^10^ However, stenting without lead extraction is undesirable. Trapping the lead between the vein wall and the stent should be avoided, since the inflexible surface of the stent can cause the lead to fracture. In addition, trapped leads cannot be percutaneously extracted in case of lead failure or device-related infection in the future.

The selection of powered sheaths (mechanical or laser sheath) is based on the circumstances of each hospital where lead extraction is performed. If there is access to both mechanical sheaths, including the Evolution RL sheath and a laser sheath, the laser-first approach is advantageous because the flexible shaft has high coaxiality with the lead, which facilitates the introduction of the laser sheath into the implanted veins. However, the laser-first approach may not be suitable for our case because of the presence of multiple leads. The significant association between multiple leads extracted per procedure and device crossover from a laser sheath to the Evolution sheath during the procedure indicates that the incidence of lead-to-lead adhesions increases if more leads are implanted, thereby requiring device crossover during lead extraction.^11^ Thus, lead-to-lead adhesions caused by multiple leads can be a barrier to laser lead extraction. Therefore, the Evolution sheath was used from the beginning of lead extraction in this study.

## Conclusion

This was a case of symptomatic lead-related venous obstruction, managed with a multidisciplinary approach. We performed percutaneous lead extraction using a tandem approach, venous stenting at the site of occlusion, and immediate implantation of a new CRT-D via the same access site in a single session. This “one-step” percutaneous intervention may be an option for this unique condition.

## Disclosures

None.

## References

[1] Vaidya V.R., Asirvatham R., Kowlgi G.N. (2022). Trends in cardiovascular implantable electronic device insertion between 1988 and 2018 in Olmsted county. JACC Clin Electrophysiol.

[2] Zecchin M., Torre M., Carrani E. (2021). Seventeen-year trend (2001–2017) in pacemaker and implantable cardioverter-defibrillator utilization based on hospital discharge database data: an analysis by age groups. Eur J Intern Med.

[3] Ferro E.G., Kramer D.B., Li S. (2023). Incidence, treatment, and outcomes of symptomatic device lead-related venous obstruction. J Am Coll Cardiol.

[4] Akhtar Z., Kontogiannis C., Elbatran A.I. (2023). Transvenous lead extraction: experience of the tandem approach. Europace.

[5] Chan A.W., Bhatt D.L., Wilkoff B.L. (2002). Percutaneous treatment for pacemaker-associated superior vena cava syndrome. Pacing Clin Electrophysiol.

[6] Gabriels J., Chang D., Maytin M. (2021). Percutaneous management of superior vena cava syndrome in patients with cardiovascular implantable electronic devices. Heart Rhythm.

[7] Sheikh M.A., Fernandez B.B., Gray B.H., Graham L.M., Carman T.L. (2005). Endovascular stenting of nonmalignant superior vena cava syndrome. Catheter Cardiovasc Interv.

[8] Riley R.F., Petersen S.E., Ferguson J.D., Bashir Y. (2010). Managing superior vena cava syndrome as a complication of pacemaker implantation: a pooled analysis of clinical practice. Pacing Clin Electrophysiol.

[9] Aldoss O., Arain N., Menk J., Kochilas L., Gruenstein D. (2014). Endovascular stent provides more effective early relief of SVC obstruction compared to balloon angioplasty. Catheter Cardiovasc Interv.

[10] Iwakawa H., Suzuki T., Terata K., Watanabe H. (2023). Successful treatment of lead-related superior vena cava syndrome in combination with transvenous lead extraction and venous stenting. J Arrhythm.

[11] Isawa T., Honda T., Yamaya K., Toyoda S., Taguri M. (2023). Associated factors and outcomes of crossover from a laser sheath to a bidirectional rotational mechanical sheath during transvenous lead extraction. J Arrhythm.

