# Supplementary material for: A “one-step” treatment for symptomatic lead-related venous obstruction using percutaneous lead extraction, venous stenting, and new device implantation
Source: HeartRhythm Case Rep. 2024 Mar 15;10(6):394–7. doi: 10.1016/j.hrcr.2024.03.004 (PMC11228062; doi:10.1016/j.hrcr.2024.03.004)
Supplement: Supplementary Material [file mmc6.docx]

**Supplementary figures titles/legends**

**
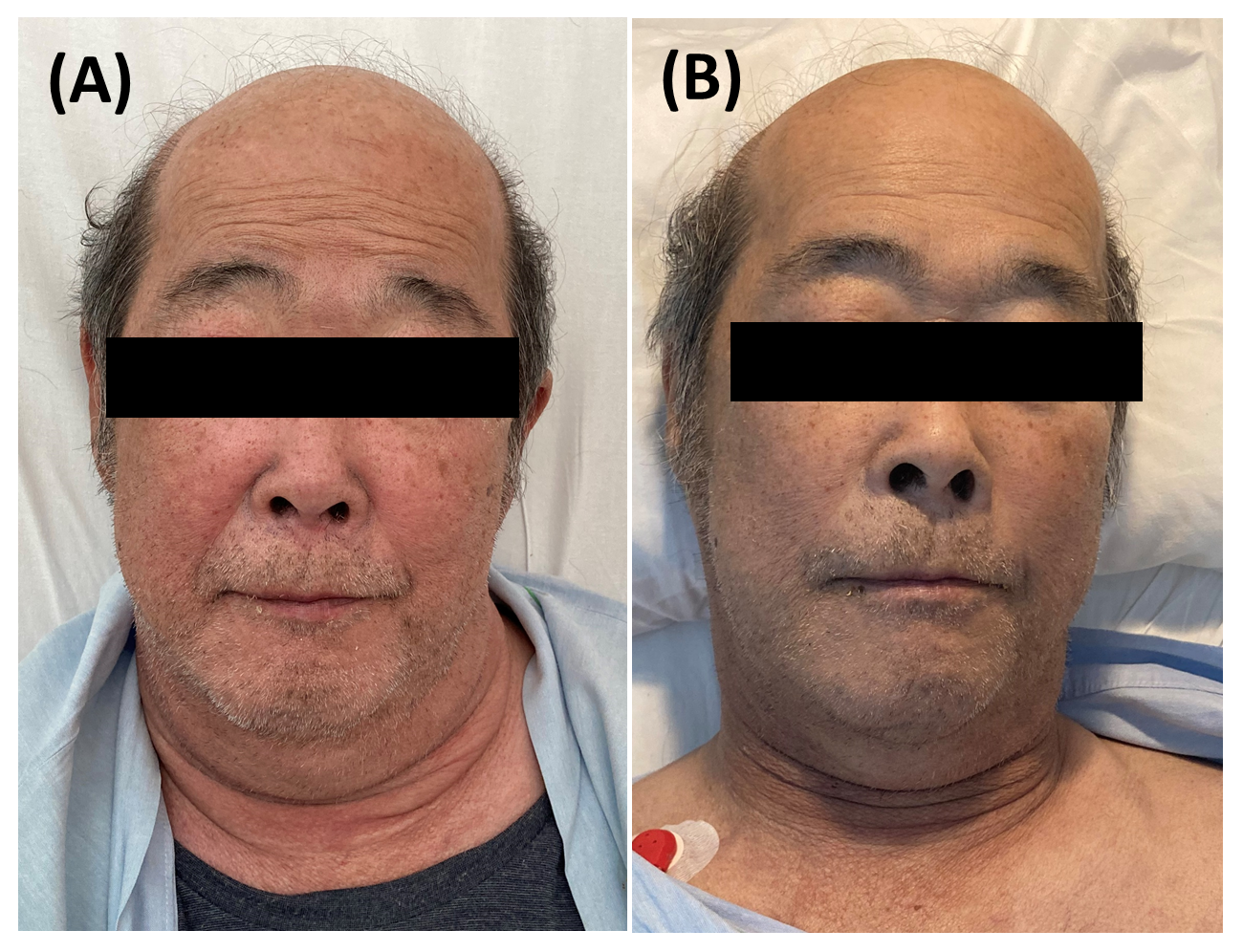
**

**Figure 1:** A comparison between front images of the patient taken before lead extraction (A) and two weeks after lead extraction with venous stenting (B). Post-lead extraction image revealing that the previously noted facial plethora and neck fullness have resolved.

**Supplementary videos titles/legends**

**Video 1:** Digital subtraction venography. Obstruction of the right brachiocephalic vein and upper superior vena cava with drainage via the azygous system.

**Video 2:** The atrial lead was completely extracted before catching the atrial lead via the femoral approach.

**Video 3:** A 9-Fr Evolution RL sheath (Cook Medical) was successfully advanced over the left ventricular lead. The femoral approach using a 13-mm Needle’s Eye Snare (Cook Medical) provided a strong rail allowing the Evolution RL sheath to advance.

**Video 4:** A 11-Fr Evolution Shortie RL sheath followed by a 11-Fr Evolution RL sheath was successfully advanced over the right ventricular lead. The femoral approach using a 13-mm Needle’s Eye Snare provided a strong rail allowing the Evolution RL sheath to advance.

**Video 5:** Post-stenting venogram. The superior vena cava demonstrated improved expansion and blood flow.
